# Problem gambling and anxiety disorders in the general swedish population – a case control study

**DOI:** 10.1007/s10899-022-10117-7

**Published:** 2022-04-05

**Authors:** Kristina Sundqvist, Peter Wennberg

**Affiliations:** 1 Department of Psychology, Stockholm University, Stockholm, Sweden; 2 Department of Public Health Sciences, Stockholm University, Stockholm, Sweden; 3 Department of Global Public Health, Karolinska Institutet, Solna, Sweden

## Abstract

Co-occurring psychiatric comorbidity is high among problem gamblers, and anxiety disorders has repeatedly been linked to problem gambling. Less conclusive, however, is the association between problem gambling and specific anxiety disorders. The aim of this study is to examine the association between problem gambling and specific anxiety disorders in subgroups of gender, age and socio-economic status (SES) in the general Swedish population. A case-control design was employed - nested in the Swedish longitudinal gambling study cohort. All anxiety disorders studied - Panic Disorder, Social Phobia, Generalized Anxiety Disorder (GAD) and Post-Traumatic Stress Disorder (PTSD), were significantly associated with problem gambling, however the pattern differed across subgroups. Social Phobia was the anxiety disorder most commonly associated with problem gambling across subgroups. The strongest associations between problem gambling and various anxiety disorders were found in participants under the age of 25, among females, and in the group with middle SES. In those groups three of the four anxiety disorders studied were significantly associated with problem gambling, with different patterns. Quite remarkably, participants under the age of 25 had three times higher risk of having had GAD compared to their controls. Efforts to prevent an escalation of either gambling or anxiety could target the presented vulnerable groups specifically.

## Background

Gambling engagement is often thought of to be on a continuum, ranging from non-gambling and recreational gambling on one end, to a psychiatric condition—gambling disorder, on the other (Volberg et al., 2015). The broader term problem gambling, applied in this study, is often used to include those that suffer significant consequences from their gambling without filling the criteria for a diagnosis (Blaszczynski & Nower, 2002; Neal, Delfabbro, & O’Neil, 2005). Prevalence rates of problem gambling vary pending on study and cultural settings, with an average across all countries of 2.3% (Williams, Volberg, & Stevens, 2012). In Sweden, 1.3% of the adult population are categorized problem gamblers, and of those 0.6% are considered disordered gamblers (Public Health Agency of Sweden, 2019).

The co-occurrence of psychiatric comorbidity in general is high among problem gamblers, and anxiety disorders has repeatedly been linked to problem gambling (Raylu & Oei, 2002; Shaffer & Martin, 2011). A meta-analysis conclude that anxiety disorders is one of the most prevalent (37.4%) psychiatric condition in population-representative samples of problem gamblers (Lorains, Cowlishaw, & Thomas, 2011). Lifetime prevalence among problem gamblers have been found to be as high as 60% (Kessler, Hwang, Labrie, et al., 2008). If studying treatment seeking problem gamblers, the numbers are even higher.

Less clear, however, is the pattern of specific anxiety disorders among problem gamblers. Studies on community samples have found the lifetime prevalence for panic disorder among problem gamblers to be between 5.1 and 21.9% (Kessler, Hwang, Labrie, et al., 2008; Petry, Stinson, & Grant, 2005), for social phobia 10.1% (Petry et al., 2005), for Generalized Anxiety Disorder (GAD) between 11.2 and 16.6% (Kessler, Hwang, Labrie, et al., 2008; Petry et al., 2005) and for Post-Traumatic Stress Disorder (PTSD) 14.8–24% (Kessler, Hwang, Labrie, et al., 2008; Moore & Grubbs, 2021; Toneatto & Pillai, 2016).

In a systematic review and meta-analysis of the prevalence of *concurrent* co-morbid psychiatric disorders among treatment-seeking problem gamblers, Dowling et al. (2015) found that the anxiety disorders with the highest weighted mean effects were social phobia (14.9%; range 5–50), GAD (14.4%; range 3.8–50), panic disorder (13.7%; range 3.8–38.9) and post-traumatic stress disorder (12.3%; range 5.0-34.2). However, the variation across studies were high, indicating a diversity in study population and methodology.

Studies examining the association between problem gambling and specific anxiety disorders have yielded diverse results. Cunningham-Williams et al. (1998) found that problem gamblers, compared to non-gamblers, were significantly more likely to have phobias (14.6% vs. 9.5%), but none of the other anxiety disorders studied. Opposite this, Petry (2005) found panic disorder (with and without agoraphobia) to be strongly related to pathological gambling, whereas the relationships between phobias and generalized anxiety disorder were weaker but still significant.

Anxiety can cause problem gambling and problem gambling can cause anxiety (Hartmann & Blaszczynski, 2018; Holdsworth, Haw, & Hing, 2012). The nature of this reciprocal effect between problem gambling and specific anxiety disorders is unclear but can, in line with the Pathway Model (Blaszczynski & Nower, 2002), be understood according to different paths. When anxiety (or other mental health issues) is present before the gambling problems, gambling can be seen as result of poor coping strategies; that is, gambling is used as way to escape emotional distress. This subtype is labeled ‘the emotionally vulnerable’ group according to Blaszczynski & Nower (2002). For this subtype, gambling behaviours may be viewed as a manifestation of maladaptive coping, with a more general underlying vulnerability involving for example an anxiety disorder. Studies have found it to more common among younger respondents to report gambling for coping reasons (Sundqvist, Jonsson, & Wennberg, 2016; Wardle, Dobbie, Kerr, & Reith, 2009). Gambling for coping reason has also been linked to more severe gambling problems (McGrath, Stewart, Klein, & Barrett, 2010) and female gender (Francis, Dowling, Jackson, Christensen, & Wardle, 2014). Alternatively, problem gambling can precede the onset of anxiety and hence can be seen as a response to gambling-related stressors, such as feelings of guilt or financial difficulties. This path is labeled the behaviorally conditioned in the Pathway model, and is characterized by the absence of premorbid sensitivity. In line with the emotionally vulnerable path, an Australian longitudinal study (Billi, Stone, Marden, & Yeung, 2014) found anxiety to be the only health condition that independently predicted the progression to high-risk gambling. Two studies have found problem gambling to predict the subsequent onset of generalized anxiety disorder and posttraumatic stress disorder (Chou & Afifi, 2011; Kessler, Hwang, LaBrie, et al., 2008), suggesting a behaviorally conditioned path. Another study found that, compared to non-gamblers, those reporting any gambling behavior at baseline were at increased risk to have any anxiety disorder (panic disorder, social and specific phobia, GAD) at follow-up (Parhami, Mojtabai, Rosenthal, Afifi, & Fong, 2014). And Blanco et al., (2015) found that childhood-onset anxiety had significant main effects in predicting lifetime gambling (but not disorder).

In the majority of studies on gambling and mental health, anxiety is treated as a homogeneous entity and is often one in a large set of risk factors analyzed. Hence, even though the link between problem gambling and anxiety appears to be well established, the evidence has been less conclusive for the relationship between problem gambling and specific anxiety disorders. This calls for more studies that disentangles the different anxiety conditions in relation to problem gambling. In addition, problem gambling, as well as anxiety, differ across subgroups. Examining strata, rather than the gambling population as a whole, might reveal subgroup specific patterns.

### Aim

The aim of this study is to examine the association between problem gambling and specific anxiety disorders in a non-clinical population. In addition, we also aimed at examining this association in different strata of the population.

## Methods

### Design

*The Swedish longitudinal gambling study* (Swelogs, 2008-), is a research program on gambling and problem gambling, managed by the Public Health Agency of Sweden (Romild, Volberg, & Abbott, 2014; The Public Health Agency of Sweden, 2013). Swelogs includes an Epidemiological track (EP) with a stratified random sample of 15,000 individuals (Romild et al., 2014) and an In-Depth track (ID) using a case-control study nested in the Swelogs cohort. Details about the data collection of the ID track has been described in detail elsewhere (Fröberg, 2015; Sundqvist & Rosendahl, 2019), and will consequently only be described briefly below.

The purpose of the ID track was to gather information about the lifetime mental health of the study participants. Individuals scoring 3 or more on the Problem Gambling Severity Index (PGSI 12 months) or on The South Oaks Gambling Screen-Revised Life Time measure (SOGS-R Life) in the EP-track was selected as cases (n = 591). The controls (n = 2400) were frequency matched to the cases based on sex and age, with a case/control ratio of 1:3. Study participants were telephone interviewed at two time points (ID1 in 2011 and ID2 in 2013) by the Centre for Psychiatry Research at Karolinska Institutet. The interviews included gambling related issues, a psychiatric diagnostic assessment, life stressors and adverse events, family and participant socio-demographic aspects. Individuals not reached by phone were sent a postal questionnaire. Socio-demographic information from official registers was linked to the data set.

### Participants

The study populations comprised of participants from the ID1 (2011), since lifetime measures for anxiety disorders was not used in ID2. The sample consists of 427 cases (34% female) and 1583 controls (35% female). See Table 1 for further sample characteristics

**Table 1:** Characteristics of cases and controls. Lifetime measures. Odds Ratios and 95% Confidence Intervals

	Case % n = 427	Control % n = 1583	OR	95% CI
**MATCHING VARIABLES**				
**Gender F/M**	35/65	34/66		
**Age M (SD)**	28.2 (13.7)	28.1 (14.7)		
**NON-MATCHING VARIABLES**				
**SES (low vs. high)**				
*Low*	27	20	1.7	1.2–2.2
*Medium*	44	45		
*High*	28	35		
**Any Depression**	33.3	20.2	2.0	1.5–2.5
**Any Alcohol Dependence**	34.3	15.9	2.8	2.1–3.6
**Any Illicit Drug Use**	7.7	3.7	2.2	1.3–3.5
**Suicidal Ideations**	21.2	11.2	2.1	1.6–2.8
**Suicidal Attempts**	6.6	3.3	2.1	1.3–3.4
**Highest (SD) PGSI score**	4.0 (3.6)	0.4 (1.0)		
**Highest (SD) SOGS score**	4.1 (2.6)	0.5 (0.8)		

*Note*: Highest mean PGSI and SOGS scores is based on each participants’ highest total score across measure points. The PGSI measures past year problems, and the SOGS both lifetime and past year

### Measures

#### Anxiety disorders

Anxiety disorders (panic disorder, social phobia, generalized anxiety disorder and posttraumatic stress disorder) was measured using subscales from the diagnostic instrument Mini International Neuropsychiatric Interview 6.0 (MINI; Sheehan et al., 1998). MINI covers six of the major and most clinically relevant anxiety disorders and has been validated in several cultural settings and the test re-test reliability of the subscales of relevance have been found to range from 0.76 to 0.93 (Lecrubier et al., 1997). The questions have the response alternatives yes or no, and interviewers follow a manual for assessment. The MINI 6.0 is based on the previous version of The Diagnostic and Statistical Manual of Mental Disorders (Rennert et al., 2014; DSM-IV-TR; American Psychiatric Association, 2000), hence PTSD is included as an anxiety disorder even though it was later moved to the stress-and trauma section (DSM-5, American Psychiatric Association, 2013). Due to time constraints of the interviews in the SWELOGS project, agoraphobia and obsessive-compulsive disorder were not included in the assessment. Other anxiety disorders such as specific phobias and separation anxiety disorder is not covered by the MINI.

#### Gambling measures

Swelogs includes both The South Oaks Gambling Screen-Revised Life Time measure (SOGS-R Life) and the Problem Gambling Severity Index (PGSI; Ferris & Wynne, 2001). The SOGS was developed to use in clinical settings among adults (Lesieur & Blume, 1987), and have been found to have satisfactory psychometric properties; test re-test reliability 0.71–0.74 and internal consistency 0.97 (Lesieur & Blume, 1987; Stinchfield, 2002). SOGS-R comprises 21 items, of which 20 dichotomous items adds up into a summary score of 0–20 points.

The PGSI was administered to respondents reporting any gambling in the past twelve months. The PGSI was developed to measure problem gambling in the general population (Ferris & Wynne, 2001), and have been found to have high internal reliability; 0.85 (Holtgraves, 2009; Orford et al., 2010). PGSI is a 9-item measure, with response alternatives from never to almost always (0–3 points per item), and with a maximum score of 27 points. Based on the sum-score, respondents are usually categorized into: non-problem gambling (0), low-risk gambling (1–2), moderate risk gambling (3–7), and problem gambling (8+) (Ferris & Wynne, 2001). In practice, to increase statistical power the categories problem gambling and moderate risk gambling are often collapsed. In the Swelogs project, as well as in this study, the categories with a score of 3–7 and 8–27 was collapsed to one category - problem gambling. Previous research has shown that this group is more likely to experience negative consequences as well as being at a greater risk of having other comorbid mental health disorders (Cox et al., 2005).

#### Other measures: socio-economic status

Socio-demographic information was gathered from official national registers. The variable socio-economic status (SES) was based on educational level and was categorized as follows; low SES - primary or lower secondary school, medium SES - upper secondary school and high SES - post-secondary or tertiary school.

### Analyses

Cases and controls were compared regarding anxiety disorders using binary logistic regression. Separate analyses were conducted with each anxiety disorder. Since the cases and controls in this study was matched based on age and gender, the analyses were stratified on those variables. Stratified analyses were also done for each SES subgroup.

Secondly, multivariate analyses were conducted to explore which anxiety disorder that best explained the variance between cases and controls within each subgroup (see Table 2). In the adjusted model, gender, age and SES were not included. However, sensitivity analyses conducted including those variables did not show any significant effect on the associations between problem gambling and any of the anxiety disorders. Lifetime measures was used for all anxiety conditions.

All participants that were interviewed in ID 1 were included in the analyses.

Data was analyzed using IBM SPSS statistics 26.

### Response rate and attrition

Of the 2400 selected participants, 1 876 were interviewed and an additional 134 responded via survey, giving a response rate of 83.8%. A larger proportion of the controls responded, compared to the cases (89.5% versus 75.5%). There were no differences in response rate across gender.

## Results

In the unstratified sample, all anxiety disorders were significantly associated with problem gambling (see Table 2), with social phobia and GAD remaining significant after simultaneously controlling for the other anxiety disorders. This pattern clearly differed when instead analyzing different subgroups of the study population. Overall, having had any anxiety disorder was associated with problem gambling in most groups except among age 25- and high SES. The weakest association between any anxiety and problem gambling was among men, and the strongest among younger (age − 25) and middle SES.

Among females, all anxiety disorders, except GAD, was significantly associated with problem gambling. The strongest association was for PTSD and problem gambling (OR = 2.5, CI = 1.3–4.8). When simultaneously controlling for the other anxiety disorders, social phobia was the only one remaining significantly related to problem gambling. Among males, social phobia was the only anxiety disorder significantly related to problem gambling (OR = 2.1, CI = 1.1–3.8), and this association remained significant when controlling for the other anxiety disorders.

In the group with participants age 24 and younger, all anxiety disorders but PTSD was significantly associated with problem gambling. GAD was most strongly associated (OR = 3.6, CI = 1.7–7.8). After simultaneously controlling for the other anxiety disorder, social phobia and GAD, but not panic disorder, remained significantly associated with problem gambling. For the group age 25 and above, social phobia and PTSD was significantly associated with problem gambling, with social phobia remaining significantly associated after adjusting for the other anxiety disorders.

The pattern of anxiety disorders differed across groups of socio-economic status. In the group with low SES the only anxiety disorder significantly associated with problem gambling was PTSD, and this was still true after controlling for the other anxiety disorder. For participants with middle SES, all anxiety disorders, but GAD, was significantly associated with problem gambling, with social phobia remaining significantly associated with problem gambling after adjusting for the influence of the other anxiety disorder. Within the group of individuals with high SES, anxiety was not at all significantly associated with problem gambling.

**Table 2:** Prevalence of anxiety disorders among different subgroups of cases and controls. Crude and adjusted odds ratio. N = 1876

Subgroup Disorder	Cases % n = 399	Controls % n = 1477	Crude OR (CI)		Adjusted OR (CI)	
**All**						
Panic Disorder	11.4	7.9	**1.5**	**(1.0-2.2)**	1.3	(0.9–1.8)
Social Phobia	8.8	4.0	**2.4**	**(1.5–3.6)**	**2.0**	**(1.3–3.1)**
GAD	4.5	2.2	**2.1**	**(1.2–3.7)**	**1.9**	**(1.1–3.5)**
PTSD	5.3	2.4	**2.3**	**(1.3-4.0)**	1.7	(1.0-3.1)
Any	22.4	13.9	**1.8**	**(1.4–2.4)**		
**Female**						
Panic Disorder	19.5	11.9	**1.8**	**(1.1-3.0)**	1.5	(0.9–2.5)
Social Phobia	13.5	5.4	**2.8**	**(1.5–5.2)**	**2.1**	**(1.1–4.2)**
GAD	6.7	3.6	2.0	(0.90 − 4.5)	1.8	(0.8–4.3)
PTSD	11.9	5.1	**2.5**	**(1.3–4.8)**	1.7	(0.9–3.6)
Any	35.6	21.1	**2.1**	**(1.3–3.1)**		
**Male**						
Panic Disorder	7.3	5.8	1.3	(0.74 − 2.2)	1.1	(0.6-2.0)
Social Phobia	6.5	3.2	**2.1**	**(1.1–3.8)**	**1.9**	(**1.0-3.6)**
GAD	3.4	1.6	2.2	(0.97 − 5.2)	2.1	(0.9-5.0)
PTSD	1.9	0.9	2.1	(0.68 − 6.2)	1.7	(0.6–5.4)
Any	15.8	10.1	**1.7**	**(1.1–2.5)**		
**Age − 24**						
Panic Disorder	10.6	6.0	**1.8**	**(1.1–3.1)**	1.4	(0.8–2.5
Social Phobia	7.4	2.7	**2.9**	**(1.5–5.6)**	**2.2**	(**1.1–4.4)**
GAD	6.0	1.7	**3.6**	**(1.7–7.8)**	**3.2**	(**1.4-7.0)**
PTSD	4.1	1.8	2.3	(0.99 − 5.3)	1.7	(0.7–4.1)
Any	20.9	10.3	**2.2**	**(1.5–3.4)**		
**Age 25-**						
Panic Disorder	12.4	10.2	1.3	(0.75 − 2.0)	1.1	(0.6–1.9)
Social Phobia	10.6	5.5	**2.0**	**1.1–3.6)**	**1.8**	(**1.9–3.4)**
GAD	2.8	2.9	0.96	(0.36 − 2.6)	0.9	(0.4–2.5)
PTSD	6.7	3.0	**2.3**	**(1.1–4.8)**	1.7	(0.8–3.3)
Any	24.3	18.3	1.4	(0.97 − 2.1)		
**Low SES**						
Panic Disorder	11.9	9.5	1.3	(0.63 − 2.7)	1.0	(0.44 − 2.1)
Social Phobia	11.8	6.1	2.1	(0.96 − 4.4)	1.3	(0.56 − 3.2)
GAD	4.9	1.4	3.8	(0.99-14.3)	3.2	(0.8-13.3)
PTSD	11.8	4.4	**2.9**	**(1.3–6.6)**	**2.7**	(**1.1–6.5)**
Any	26.7	16.3	**1.9**	**(1.1–3.2)**		
**Middle SES**						
Panic Disorder	12.9	7.2	**1.9**	**(1.1–3.3)**	1.5	(0.89 − 2.7)
Social Phobia	9.2	3.2	**3.1**	**(1.6-6.0)**	**2.6**	**(1.3–5.3)**
GAD	3,5	1.5	2.3	(0.83 − 6.5)	2.0	(0.71 − 5.9)
PTSD	4.0	1.4	**3.0**	**(1.1–8.3)**	1.7	(0.57 − 5.2)
Any	22.9	10.2	**2.5**	**(1.6–3.8)**		
**High SES**						
Panic Disorder	7.1	8.1	0.86	(0.39 − 1.9)	0.72	(0.3-1.7)
Social Phobia	6.3	3.8	1.7	(0.70 − 4.2)	1.9	(0.8-4.8)
GAD	6.2	3.6	1.7	(0.69 − 4.1)	1.7	(0.7-4.1)
PTSD	1.8	2.6	0.68	(0.15 − 3.1)	0.68	(0.1-2.8)
Any	17.1	16.7	1.0	(0.60 − 1.8)		

*Note*: GAD = Generalized Anxiety Disorder, PTSD = Post Traumatic Stress Disorder. In the adjusted model each anxiety disorder is adjusted for the other anxiety disorders

## Discussion

In this study, the associations between problem gambling and specific anxiety disorders were examined in different subgroups, using a case control design with a sample from the general Swedish population. Overall, having had any anxiety disorder was significantly more common among the cases compared to their controls in most subgroups, except for the group aged 25 and over, and in the group with high SES. The magnitude of the associations varied with the lowest among males (70% greater risk compared to their controls) and the highest for middle SES (150% greater risk then their controls).

After also controlling for the other anxiety disorders, social phobia was the most common anxiety disorder to be associated with problem gambling across groups. This was true for both men and women, in both age groups, but only for the middle SES group. GAD was also associated with problem gambling in the whole study population, but this association was only statistically significant in one of the subgroups - younger (age − 24). Younger had three times higher risk of having had GAD compared to their controls, after controlling for the other anxiety disorders. PTSD was only significantly associated with problem gambling in the group with low SES. Panic Disorder was the anxiety disorder with the weakest association with problem gambling.

Previous studies on community-based samples have generally found stronger associations between each anxiety disorder and problem gambling. This can likely be explained by the fact that our study includes gamblers with mild problems (PGSI 3+), whereas other studies have mainly focused on groups with more severe gambling problems. For example, both Petry et al. (2005) and Kessler et al. (2008) used five out of ten DSM-IV criteria as a cut of for problem gambling, yielding a sample of gamblers with more severe gambling problems compared to our study sample. This pattern is also found in studies of the association between substance use disorders and anxiety disorders, where alcohol- and drug dependence is significantly associated with several anxiety disorders whereas for the group with milder symptoms (abuse) the associations are weaker (Smith 2012; Smith & Book, 2008).

Further, in contrast to the results in our study, Petry et al. (2005) found social phobia to be the anxiety disorder with the weakest association to problem gambling. In addition, in their study panic disorder, which in our study had the lowest (and non-significant OR), had the strongest association to problem gambling. There are several possible explanations for this discrepancy, such as different study populations, methods and measures used. Another reason for the difference found might be the increased use of online gambling during the years since the study of Petry et al. (2005) was conducted. This in turn might attract individuals that avoid public locations such as land-based casinos. However, our results are in line with Brooker et al. (2009), who also found problem gambling to be associated with social phobia, but not with panic disorder. Brooker et al. (2009) used the same measure and cut of for problem gambling as in this study.

In addition, the results from our study differ slightly from previous research where low SES repeatedly has been associated with a greater risk of mental health issues in general (Hudson, 2005; Hudson & Roth, 1988; Kivimäki et al., 2020). Specific to gambling, Maas et al. (2016) found that the magnitude of the relationship between anxiety disorders and problem gambling severity varied significantly depending on whether a person were of high or low SES, with the strongest association among the low SES group. Even though the group with high SES had the weakest association also in our study, the association between any anxiety and problem gambling was stronger among the group with middle SES, then in the low SES group. In addition, the most common anxiety disorder among the problem gamblers across groups in this study, social phobia, was not at all significantly related to gambling in the low SES group. PTSD, however, was significantly associated with problem gambling only among the group with low SES. However, those results should be interpreted with caution since the low SES group is the smallest subgroup studied (n = 401).

A major strength in this study is the use of a representative sample from the general population, as well as the inclusion of problem gamblers ranging in severity from mild to severe, which mirrors the actual gambling situation in society and generates more generalizable results compared to results from studies using treatment seeking samples. Another strength is that the assessment of anxiety disorders was based on clinical interviews rather than self-assessment measures. In addition, the stratified analyses made it possible to reveal patterns specific to different subgroups. The participation rate was 84% which can be regarded as satisfactory in this context.

A limitation with this study is the fact that excessive gamblers tend to go in and out of gambling problems, which may affect the groups of cases and controls. Since group allocation is defined based on PGSI or SOGS scores about a year before the clinical interview, there is a risk that cases include problem gamblers in remission, and that the control group includes a few problem gamblers. This, however, is to some extent taken care of by only using life-time measures in the analyses. Another limitation is that the SWELOGS interviews only covers four anxiety disorders. Further, due to the nature of the study design, the results can only be interpreted as associations rather than causal relationships. Finally, there is a risk that the adjusted model might be over adjusted, due to the fact that the anxiety disorders studied are interrelated. For this reason, the crude odds ratios are of interest as well.

## Conclusions

All anxiety disorders studied were significantly associated with problem gambling, however the pattern differed across subgroups. Social phobia was the anxiety disorder most commonly associated with problem gambling across subgroups. For participants under the age of 25, problem gambling was strongly associated with GAD. In the groups of females, younger, and participants with middle SES more anxiety disorders were significantly associated, and those associations were also stronger, than in the other subgroups. Even mild problem gambling is associated with anxiety, especially in some sub-groups. Preventive interventions could target those sub-groups specifically.
